# Bioremediation of Perfluoroalkyl Substances (PFAS) by Anaerobic Digestion: Effect of PFAS on Different Trophic Groups and Methane Production Accelerated by Carbon Materials

**DOI:** 10.3390/molecules27061895

**Published:** 2022-03-15

**Authors:** Ana Rita Silva, Maria Salomé Duarte, Maria Madalena Alves, Luciana Pereira

**Affiliations:** 1 CEB, Centre of Biological Engineering, University of Minho, 4710-057 Braga, Portugal; ana.rita.silva@ceb.uminho.pt (A.R.S.); salomeduarte@ceb.uminho.pt (M.S.D.); madalena.alves@deb.uminho.pt (M.M.A.); 2 LABBELS—Associate Laboratory, 4800-122 Braga/Guimarães, Portugal

**Keywords:** anaerobic processes, activated carbon, carbon nanotubes, sewage sludge, specific methanogenic activity, perfluorooctanoic acid, perfluorooctane sulfonate

## Abstract

Per- and polyfluoroalkyl substances (PFAS) are recalcitrant pollutants which tend to persist in soils and aquatic environments and their remediation is among the most challenging with respect to organic pollutants. Anaerobic digestion (AD) supplemented with low amounts of carbon materials (CM), acting as electron drivers, has proved to be an efficient process for the removal of organic compounds from wastewater. This work explores the impact of PFAS on different trophic groups in anaerobic communities, and the effect of carbon nanotubes (CNT), activated carbon (AC), and oxidized AC (AC-HNO_3_), as electron shuttles on the anaerobic bioremoval of these compounds, based on CH_4_ production. The inhibition of the specific methanogenic activity (SMA) exerted by perfluorooctanoic acid (PFOA) and perfluorooctane sulfonate (PFOS), at a concentration of 0.1 mg L^−1^, was below 10% for acetoclastic and below 15%, for acetogenic communities. Hydrogenotrophic methanogens were not affected by the presence of PFAS. All CM reduced the negative impact of PFAS on the CH_4_ production rate, but AC was the best. Moreover, the methanization percentage (MP) of sewage sludge (SS) increased 41% in the presence of PFOS (1.2 g L^−1^) and AC. In addition, AC fostered an increase of 11% in the MP of SS+PFOS, relative to the condition without AC. AC promoted detoxification of PFOA- and PFOS-treated samples by 51% and 35%, respectively, as assessed by *Vibrio fischeri* assays, demonstrating the advantage of bringing AD and CM together for PFAS remediation.

## 1. Introduction

PFAS, including per- and polyfluoroalkyl substances, are organofluorine compounds highly used in several industrial sectors for applications such as oil production, refrigerators, firefighting foams, textiles, and stain resistance products, among others [1,2]. These compounds are known to pose environmental and human health risks due to their wide distribution, recalcitrant behavior, bioaccumulation tendencies, and potential toxicological effects [1,3]. The environmental remediation of PFAS from water and soils is challenging since their fate in the environment is still not yet completely known, and they are extremely recalcitrant to conventional wastewater treatments. In this sense, developing effective methods for identifying PFAS, and processes for treating matrices containing them, is a topic of growing urgency [4].

Among PFAS, perfluorooctanoic acid (PFOA) and perfluorooctane sulfonate (PFOS), which have been widely manufactured and can also be formed via degradation of precursors including fluorotelomer-based compounds such as alcohols, sulfonates, and amides, among others [5,6,7], are the most frequently present in many environmental settings and are of increasing concern [3,8,9]. These compounds are often released in large quantities directly into the environment and end up in water courses [2,3]. Regarding wastewater, on average 638.2 ng L^−1^ and 465.4 ng L^−1^ of PFOA and PFOS, respectively, have been detected worldwide [9]. However, in sewage sludge (SS), these compounds tend to accumulate and PFOS was detected at concentrations up to 7304.9 ng g^−1^ of dry weight, while the highest concentration of PFOA was 241 ng g^−1^ of dry weight [9].

The carbon–fluorine (C–F) bond is the strongest single covalent bond in organic chemistry [4,9,10]. Hence, biodegradation of PFAS compounds is difficult and usually limited to molecules, or regions on the molecules, that are not fully fluorinated such as fluorobenzene, fluoroacetate, perfluorohexylethanol, and perfluorohexylsulfonate [4,10,11]. Additionally, the lack of C-H bonds in perfluorinated compounds such as PFOA and PFOS makes them more difficult to degrade [1,12].

Although biodegradation of C_2_ organohalides is well known, only few studies have reported the microbial reductive defluorination of complex perfluorinated compounds [6,8,9,11,13]. Reductive defluorination of PFOA and PFOS by pure and enrichment cultures of *Acidimicrobium* sp. strain A6, oxidizing ammonium while reducing iron (III), or using hydrogen as the electron donor, was reported by Huand and Jaflé [4]. In this study, up to 60% of PFOA and PFOS were removed, after 100 days of incubation, while total fluorine remained constant. More recently, Yu et al. [1] reported the cleavage of the C−F bond for two C_6_ PFAS by an organohalide-respiring microbial community obtained from a well-characterized dechlorinating enrichment culture, using lactate as an electron donor. PFAS were the sole electron acceptors in the medium and the reductive defluorination occurred by the release of F^−^.

In their work, Li et al. [8] found that the concentration of polyfluoroalkyl substances absorbed/desorbed on sludge decreased due to the breakdown of solids during AD and that the sludge resulting from the AD process was a significant source of perfluoroalkyl substances, which resulted from the digestion of polyfluoroalkyl substances. Indeed, anaerobic bioprocesses have been reported as efficient in removing recalcitrant compounds [14,15,16,17] and increasing the sorption capacity of hydrophobic contaminants [17,18]. However, the low reaction rates of the anaerobic biotransformation of recalcitrant compounds, largely due to electron transfer limitations, may be a barrier to their application [19]. This can be surpassed by applying redox mediators (RM), which can be reversibly oxidized and reduced, so acting as electron carriers in multiple redox reactions in anaerobic processes, increasing the overall reaction rates [20]. For instance, the reduction rates of dyes, aromatic amines, and pharmaceutical compounds were significantly improved by the application of low concentrations of different carbon materials (CM) as RM [20,21,22,23], such as activated carbon (AC) [24,25,26] and carbon nanotubes (CNT) [14,15,22,25]. In addition, unlike soluble RM which comes out with the treated effluent, therefore being an additional chemical compound in treated effluents, and at the same time needing to be fed to the reactors continuously, CM have the particularity of being easily retained in the reactors and not needing to be fed continuously [27].

CM also exhibit excellent properties such as a large specific surface area, different pore size (micro, meso, or macropores), high mechanical strength, and the possibility of incorporating functional groups on their surface, aimed at tailoring them to specific applications [14] which make them exceptional RM and also excellent adsorbents [21,28]. Recent studies have reported the removal of PFAS by adsorption [29,30]. Strong anion-exchange sorbents seem to be more efficient for the removal of both long- and short-chain PFAS. Furthermore, the adsorption capacity of long-chain PFAS is higher than short-chain PFAS [29,31,32]. Zhang et al. [33] recently reported sorption of PFAS on softwood-derived biochar and granular AC (GAC). GAC demonstrated a higher PFAS sorption capacity than biochar (43 and 39.6%, respectively), reaching the sorption equilibrium within 3–24 h, while biochar required 12–48 h. Furthermore, sorption of PFAS increased with a decrease in pH [33]. The sorption capacity of PFAS also depends on their chain length, which justifies the higher sorption capacity for PFOS than PFOA [29,31,32]. 

The development of effective biological processes requires knowledge of the toxicological effects of these compounds on microbiological communities. Likewise, toxicological evaluation before and after treatment is crucial in order to achieve not only degradation but also detoxification. However, the development of effective biological processes for the degradation and detoxification of PFAS-contaminated effluents is a research topic that is still at an early stage. 

In this study, the feasibility of applying AD for the treatment of waste/wastewater contaminated with PFAS, using PFOA and PFOS as model compounds, is addressed: firstly, evaluating the potential toxic effect of these compounds on the different trophic groups from anaerobic microbial communities, and then assessing the effect of different CM, specifically a commercial pristine AC, a tailored AC by HNO_3_ oxidation (AC-HNO_3_), and CNT, as RM of the anaerobic bioprocess. The potential detoxification of the samples by the proposed anaerobic bioprocess was assessed by the standard bioassay using *Vibrio fischeri*. Further, the biodegradability of SS contaminated with PFAS in the presence AC was evaluated. To the authors’ knowledge, there are no previous studies on the application of CM as RM in the anaerobic removal of PFAS from wastewater and SS. Thus, this work is a starting point for the application of these processes in the remediation of these recalcitrant compounds.

## 2. Results and Discussion

### 2.1. Toxicity of PFAS towards Different Anaerobic Trophic Groups

The specific methanogenic activity (SMA) of the anaerobic microbial community was assessed by measuring the CH_4_ production rate and was expressed in the volume of methane produced at standard temperature and pressure (STP) conditions per mass unit of volatile solids (VS) of inoculum and time (mL·g^−1^·d^−1^). In the control assays, conditions without PFAS, the highest SMA was obtained for H_2_/CO_2_, being (533.4 ± 10.0) mL·g^−1^·d^−1^. For acetate and VFA, the SMA was (436.3 ± 21.6) mL·g^−1^·d^−1^ and (272.7 ± 18.4) mL·g^−1^·d^−1^, respectively (Table 1). The results demonstrate that these anaerobic trophic groups are present and active on the anaerobic community used in the assays. 

In the presence of 0.1 mg L^−1^ of PFAS, a higher concentration than that usually found in WWTP [9,29], the SMA was slightly affected by both PFOA and PFAS (Table 1). The inhibition of acetoclastic methanogens exerted by PFOA and PFOS was statistically similar, (6.6 ± 5.0)% and (4.4 ± 2.4)%, respectively, while the activity of acetogenic bacteria inhibited by PFOA (7.9 ± 4.8)% and PFOS (14.8 ± 5.1)%, was also statistically similar. Hydrogenotrophic methanogens were not affected by this concentration, nor by higher concentrations of PFAS, since the low inhibition extent observed may be considered negligible. 

Acetogens were the most sensitive microbial group to which higher SMA inhibition was observed at all tested concentrations, compared with the other microbial groups. This group was more affected by PFOS, with an increase in inhibition up to (30.4 ± 2.4)%, with 80 mg L^−1^, with these differences statistically significant (*p* < 0.05). In contrast, the inhibition of acetoclastic methanogens for concentrations ranging from 1 to 25 mg L^−1^ of PFOA was slightly higher (from ≈7.5 to 17%) than in the presence of 1 to 20 mg L^−1^ of PFOS (from ≈6.6 to 13%). However, for 80 mg L^−1^, PFOS exhibited a greater toxic character towards the acetoclastic community (≈25%) than PFOA at 100 mg L^−1^ (15%). 

Fitzgerald et al. [2] reported that PFOS at a concentration of 50 mg L^−1^ was inhibitory to mixed anaerobic cultures from a seed digester, using sludge as electron donor. In this study, in the first 3 days of assay, a significantly reduction of the CH_4_ production rate by the action of PFOS was observed, compared with the control without PFAS. Additionally, in the control assay, a CH_4_ production rate of approximately 1.5 mL day^−1^ was obtained, while when PFOS were present, the CH_4_ production rate was lower than 0.5 mL day^−1^ [2]. On the other hand, PFOA did not affect the CH_4_ production rate which was similar to the control [2]. Indeed, there is some controversy about the toxicity of PFOS, as several authors reported toxicity of PFOS while others did not observe toxic effects towards anaerobic cultures [2,34,35], stating that PFOS toxicity is variable according to the microbial community [34,35,36,37]. Regarding the results obtained in the present study, it can be concluded that the anaerobic communities were not severely affected by these compounds in the tested concentrations and, therefore, the application of the AD for the treatment of wastewater or waste, such as sewage sludge (SS), contaminated with PFAS seems feasible.

### 2.2. Evaluation of the Effect of PFAS and CM on CH_4_ Production from VFA 

**Figure 1:**
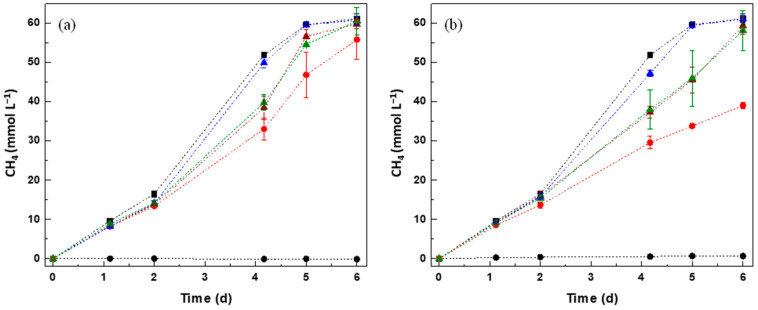
Effect of PFOA (**a**) and PFOS (**b**) on CH_4_ production from the anaerobic degradation of VFA, in the presence of CM. Blank assays (●), Controls—biotic assays without PFAS (■), biotic assays with PFAS (●), biotic assays in the presence of PFAS and: CNT (▲), AC (▲), or AC-HNO_3_ (▲).

In order to assess the possible influence of CM in reducing the toxicity of PFAS, a second assay was performed by using a mixture of VFA, 0.1 mmol L^−1^ of PFAS (50 mg L^−1^ of PFOA and 40 mg L^−1^ of PFOS), and different CM at a concentration of 0.1 g L^−1^. The activity of the anaerobic microbial community was assessed in the biological assays by measuring the VFA consumption coupled to methane (CH_4_) production (Figure 1, Table 2 and Table S1). VFA are converted to CH_4_ in several sequential reactions, where propionate and butyrate are initially converted to acetate, and to H_2_ and CO_2_ by acetogens (acetogenesis), which are further converted to CH_4_ and CO_2_ (methanogenesis) by acetoclastic and hydrogenothrophic methanogens, respectively [38]. In this experiment, VFA were totally consumed by the anaerobic granular sludge (AGS) in the absence of PFAS (Figure 1 and Table S1, see Supplementary Materials). Additionally, the CH_4_ obtained in the final of the assays is concordant with that expected by the conversion of VFA to CH_4_ (Table S1).

The presence of PFOA and PFOS decreased the methane production rate to (36.0 ± 5.5)% and (46.3 ± 1.6)%, respectively (Table 2). The greater negative impact of PFOS than PFOA on VFA consumption is in accordance with the toxicity observed in SMA assays performed in this study. Nevertheless, when CM were applied in the anaerobic process, the negative effect of PFAS was significantly reduced. For PFOA, in all the tested conditions in the presence of CM, acetate, propionate, and butyrate were completely consumed within 6 days, demonstrating the effect of CM on accelerating the reactions rates (Table S1). The inhibition of CH_4_ production decreased from (36.0 ± 5.5)% to (15.3 ± 7.2)%, (2.4 ± 2.0)%, and (14.2 ± 2.1)% in the presence of CNT, AC, and AC-HNO_3_, respectively. Regarding the assays with PFOS, propionate was completely converted in all of the conditions after 6 days, but ≈56% of butyrate and ≈59% of acetate were still present in the condition without CM, and ≈2% of butyrate and ≈51% of acetate were still present in the assay with AC-HNO_3_ (Table S1). Based on these results, CNT and AC were superior in promoting the VFA conversion to CH_4_ in the presence of PFOS. AC was the best-performed material at reducing the negative impact of PFAS on methanogenesis. The presence of AC reduced the inhibition of the CH_4_ production rate from (46.3 ± 1.6%), in its absence, to only (3.5 ± 1.3)%, which corresponds to a ≈13-fold rate improvement, with these differences statistically significant (*p* < 0.05).

On blank assays without a substrate, vestigial concentrations of CH_4_ were obtained (Figure 1), and in abiotic controls, no CH_4_ was obtained (data not shown). 

The specific chemical and surface properties of the different CM influence CH_4_ production (Table 3). CNT are mesoporous nanomaterials, presenting a *S*_BET_ of 201 m^2^ g^−1^ and *Vp* of 0.416 cm^3^ g^−1^. On the other hand, AC is a microporous material, with a *S*_BET_ of 1002 m^2^ g^−1^ and *Vp* of 0.525 cm^3^ g^−1^. The functionalization of AC by oxidative treatment with HNO_3_, resulting in AC-HNO_3_, promoted a slight decrease of these two parameters (Table 3) and also the incorporation of a greater amount of surface oxygen-containing groups [14,21,39]. Higher *S*_BET_ and *Vp* make CM good adsorbents for compounds such as PFAS, removing them from the medium and consequently reducing their negative impact [29,32,33]. Recently, Zhang et al. [33] studied the sorption of PFAS on GAC and softwood-derived biochar. Based on the obtained Langmuir sorption isotherms, GAC showed a higher sorption capacity for PFOS (123.5 µmol g^−1^) than for PFOA (86.2 µmol g^−1^).

Another important factor in the removal of pollutants from water is the surface chemistry of the CM reflecting the basicity/acidity character, which is represented by its pH_PZC_. In this sense, by knowing the CM´s pH_PZC_ and the pKa of the compounds, the prediction of the interactions between the CM, microorganisms, and pollutants will be facilitated [21,40,41]. CNT, AC, and AC-HNO_3_ applied in this study have a pH_PZC_ of 6.6, 8.4, and 4.1, respectively, which means that at the medium pH of 7, CNT present nearly neutral charge, AC positive charge, and AC-HNO_3_ negative charge [42]. On the other hand, PFOA has a pKa of 2.8 [43] and PFOS of 3.27 [44], so at neutral pH they are in the anionic form. It is expected that electrostatic interaction between CM and PFAS will be facilitated by opposite charges, promoting higher adsorption and, consequently, higher electron transfer. AC is the CM with high *S*_BET_ and *Vp*, as well as a favorable pH_PZC_, so, theoretically, the most favorable among the CM evaluated for the removal of PFAS [42,45]. In addition, the adsorption capacity of AC was demonstrated to be 440.11 and 426.49 (mg PFAS/g-adsorbent), for PFOS and PFOA, respectively [29]. This indicates that the adsorption of PFAS in the present study could have been total for PFOS and almost total for PFOA, since the dosage of PFAS per gram of AC used was 400 and 500 mg g^−1^, respectively. On the other hand, AC-HNO_3_ presents negative surface charge at pH of 7, with repulsive interactions expected, explaining the worst results obtained with this CM compared with the pristine AC. 

Furthermore, CM have been reported as efficient RM in the anaerobic removal of organic pollutants such as dyes, aromatic amines, and pharmaceuticals, significantly improving the reaction rates, with, in some cases, the biological reduction only taking place in the presence of the CM [20,42]. Thus, the improving results observed with CM (Figure 1, Table 2) may result from simultaneous adsorption and degradation mechanisms occurring in the system [15,22,42]. Better adsorption will approximate the PFAS to CM, promoting the electron transfer [15]. In the same sense, the AGS used has a negative charge that also favors the proximity to AC [15].

Although the reductive defluorination of PFAS showed as energetically favorable [46], there is little literature on the biological defluorination of these compounds [4,8]. Recently, Yu et al. [1] reported the cleavage of the C−F bond in the C6 of PFAS via reductive defluorination, as demonstrated by the release of F^−^ and the formation of the corresponding product. The anaerobic microbial community caused a reductive defluorination of PFAS when lactate was used as an electron donor, and the PFAS was the electron acceptor, resulting in a defluorination degree of 11% [1]. In addition, Huang et al. [4] reported defluorination of PFOA and PFOS by incubating *Acidimicrobium* sp. strain A6 with hydrogen as a sole electron donor. Up to 60% of PFOA and PFOS were removed, as observed after 100 days of incubation, while total fluorine remained constant [4]. 

Accordingly, from our assays it is possible to expect that adsorption and degradation of PFAS could have occurred simultaneously. However, the occurrence of defluorination was not assessed and requires further investigation [15].

### 2.3. Toxicity Assessment towards V. fischeri

The toxicity of the samples before and after the biological anaerobic treatment was assessed by the standard bioassay using *Vibrio fischeri* as a biosensor [48,49], with the results of the extent of luminescence inhibition (INH) shown in Table 4. The initial PFOA and PFOS solutions, at concentrations of 50 mg L^−1^ and 40 mg L^−1^, led to an INH of (63.3 ± 0.4)% and (58.3 ± 7.3)%, respectively, being considered toxic towards this microorganism [50,51]. In contrast, other authors have reported that PFOS exerts greater toxicity than PFOA on freshwater organisms [52,53]. Additionally, 50% of the lethal concentration (LC_50_) towards *Moina macrocopa* of 17.95 mg L^−1^ and 199.51 mg L^−1^, for PFOS and PFOA, respectively, was described by Ji et al. [53]. A LC_50_ of 25 mg L^−1^ for PFOS and 500 mg L^−1^ for PFOA was obtained with *Daphnia magna* [52,53].

With the exception of AC-HNO_3_ for PFOS treatment, in the presence of CM, the toxicity of the medium after the biological treatment of PFOA and PFOS was reduced. Regarding PFOA, the toxicity of the samples decreased after the biological treatment to (49 ± 0.4)%, (31 ± 4.9)%, and (37 ± 2.5)% in the assays with CNT, AC, and AC-HNO_3_, respectively. In the treatment of PFOS model wastewater, (40 ± 6.4)%, (38 ± 4.4)%, and (58 ± 4.6)% of INH was obtained with CNT, AC, and AC-HNO_3_, respectively (Table 4).

The best detoxification results were obtained for the treated media in the presence of AC, which promoted 51% of detoxification for PFOA and 35% for PFOS samples, followed by CNT and AC-HNO_3_. These results are consistent with those obtained in the biological anaerobic treatment (Figure 1, Table 2), where AC was the CM with the best performance in reducing the negative impact of PFAS on CH_4_ production. The INH still observed in the samples collected after anaerobic treatment may be related to the fraction of PFAS still existing in solution after treatment, or due to the possible by-products formed by the anaerobic degradation of these compounds [1,10]. The formation of by-products in biotic assays is supported by the lower toxicity observed in the abiotic assay. Since CM are good adsorbents for organic and inorganic compounds [21,39], in abiotic conditions, adsorption may be the predominant removal mechanism occurring [15], with the lower toxicity observed maybe resulting from the lower concentration of PFAS in solution. It is noteworthy that, although the presence of CM could also contribute to the final toxicity of the treated solution, previous results showed that the toxicity for the concentration used, 0.1 g L^−1^, is considered negligible [14,15,42].

The possible contribution of the anaerobic medium, and of the metabolites formed during the anaerobic process, to the toxicity of the treated solution was also assessed. However, the INH obtained for the medium itself, (4.9 ± 0.9)%, and for the control assay, (15 ± 6.7)%, is considered negligible [15,54].

### 2.4. Biomethane Production from Sewage Sludge Contaminated with PFAS in the Presence of AC 

Anaerobic biodegradability assays were performed to evaluate the biochemical methane potential (BMP) of SS contaminated with PFAS. The BMP of the control assay with cellulose corresponded to (90 ± 3)% of the methanization percentage (MP) expected only from cellulose degradation (Figure S1, Table 5). This control validated the BMP test since the criterion of 80% < MP < 100% was fulfilled.

**Figure 2:**
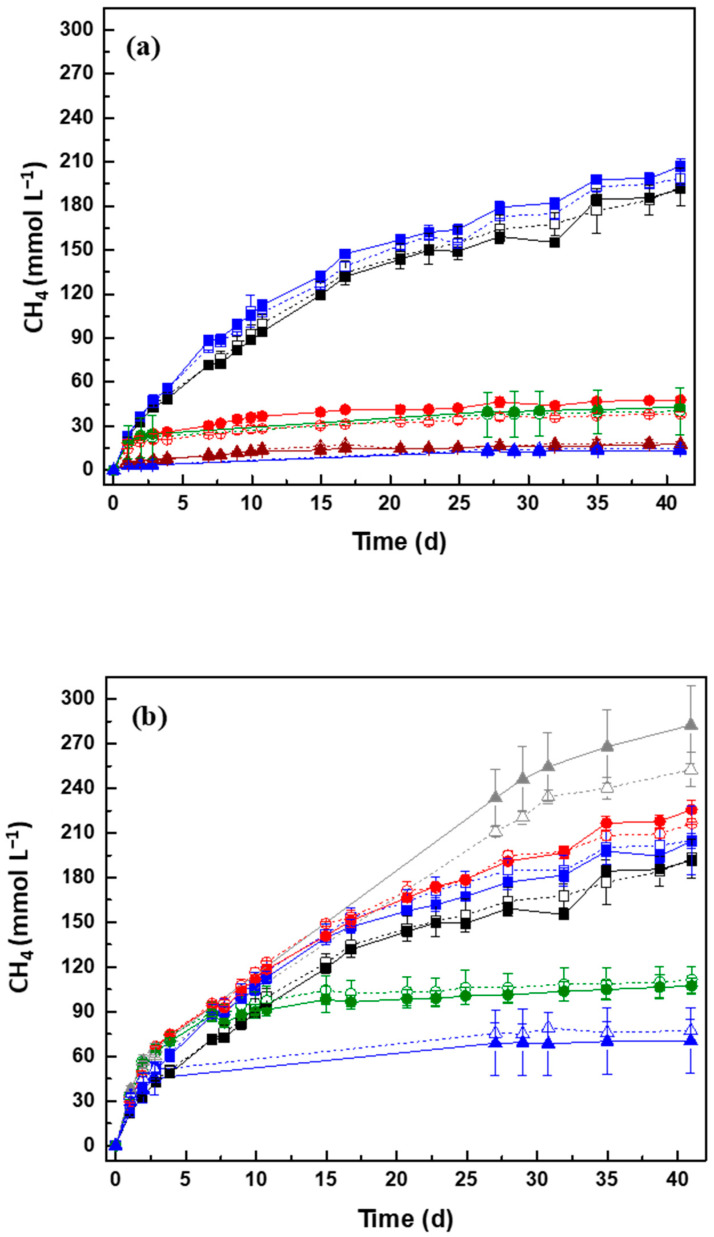
Biomethane production from sewage sludge (SS) contaminated with PFOA (**a**) and PFOS (**b**) in the presence and absence of activated carbon (AC) at 0.1 g L^−1^. Assays were conducted with SS without PFAS (□) and SS without PFAS and in the presence of AC (■). The effect of PFAS on the BMP of SS was tested for PFOA concentrations of 0.1 g L^−1^ (□), 1 g L^−1^ (○), 1.6 g L^−1^ (○), 2 g L^−1^ (Δ), and 3.4 g L^−1^ (Δ), and the effect of AC on the BMP of SS with PFOA at 0.1 g L^−1^ (■), 1 g L^−1^ (●), 1.6 g L^−1^ (●), 2 g L^−1^ (▲), and 3.4 g L^−1^ (▲). The effect of PFOS (b) was also assessed for concentrations of 0.1 g L^−1^ (□), 1 g L^−1^ (○), 1.2 g L^−1^ (Δ), 1.6 g L^−1^ (○), and 3.5 g L^−1^ (Δ), and the effect of AC on the BMP with PFOS at 0.1 g L^−1^ (■), 1 g L^−1^ (●), 1.6 g L^−1^ (●), 2 g L^−1^ (▲), and 3.5 g L^−1^ (▲).

The cumulative methane production during the anaerobic biodegradability assay is shown in Figure 2. No lag phases were observed in the methane production in all assays, demonstrating that the presence of PFAS did not affect the initial CH_4_ production (Figure 2). In addition, the residual CH_4_ produced from the inoculum was negligible (~3.08 mmol L^−1^).

PFAS dissimilarly affected the BMP of the SS in concentration comprised between 0.1 and 3.5 g L^−1^. A decrease in CH_4_ production was observed with an increase of PFOA concentration. PFOA did not affect the biodegradability of SS only when the concentration was 0.1 g L^−1^, since CH_4_ production was statistically similar with the assay of the BMP only with SS (Figure 2, Table 5). At this concentration, the MP was (41.0 ± 1.1)%, while the methanization only with SS was (42.0 ± 3.0)% (Table 5). For concentrations ranging from 1 g L^−1^ to 3.4 g L^−1^, the toxicity of PFOA increased affecting the anaerobic communities and the BMP was lower than that obtained in assay only with SS (Figure 2, Table 5).

Regarding PFOS, the biodegradability of the SS was not negatively affected for concentrations ranging from 0.1 to 1.2 g L^−1^_._ Furthermore, at this range, CH_4_ production increased with the increase of PFOS concentration, indicating that this compound may potentiate the digestion of SS or contribute to CH_4_ production. The MP of SS increased from (42.8 ± 3.8) to (52.8 ± 2.4)% and the BMP from (227 ± 20) to (280 ± 13) L·kg^−1^, with these differences statistically significant (*p* < 0.05). This positive effect of PFOS on BMP was more evident at a concentration of 1.2 g L^−1^, when an increase of (25 ± 6)% on the MP of SS was obtained (Table 5). On the other hand, for concentrations ≥ 1.6 g L^−1^, the toxic effect of PFOS towards the anaerobic community is strong, and the MP, and therefore the BMP, is lower than that obtained in assay only with SS (Figure 2, Table 5). An inhibition of (46 ± 4)% and (65 ± 3)% on the MP of SS was obtained for 1.6 and 3.5 g L^−1^ of PFOS, respectively.

AC had a positive effect on the BMP from SS containing PFAS. In concentrations ranging from 0.1 to 1.6 g L^−1^ of PFOA (Figure 2a, Table 5), an increase in the methanization of SS was observed, compared to the assays in the absence of AC. However, due to the greater toxicity of PFOA, the AC beneficial effect was more evident in PFOS assays (Figure 2b, Table 5). AC stimulated CH_4_ production for concentrations of 0.1 g L^−1^, 1.0 g L^−1^, and 1.2 g L^−1^ of PFOS, with its effect more pronounced also at 1.2 g L^−1^. At this concentration, despite the statistical similar values, AC improved the MP from (52.8 ± 2.4)% to (59.1 ± 6.2)% and the BMP from (280 ± 13) to (313 ± 33) L·kg^−1^ (Table 5), representing an increase of approximately 11%, relative to the corresponding assay without AC. The presence of AC on the SS assay (SS+AC) without PFAS did not influence the digestion of the SS (Figure 2, Table 5).

CH_4_ production of (205 ± 5) mmol L^−1^, (226 ± 6) mmol L^−1^, and (283 ± 26) mmol L^−1^, was obtained for 0.1, 1, and 1.2 g L^−1^ of PFOS, respectively (Figure 2b). These results represent an increase of 1.07 and 1.12-fold of CH_4_ production for 1 and 1.2 g L^−1^ by the action of AC, while with 0.1 g L^−1^ of PFOS, CH_4_ production in the presence and absence of AC was similar. Furthermore, after 41 days of assay, in the presence of AC, an increase of approximately 41% in CH_4_ production from SS contaminated with 1.2 g L^−1^ PFOS was observed, compared to the assay without PFAS (only SS) (Table 5). 

The obtained results are an indication that AC promoted the biodegradability of SS contaminated with PFAS. Nevertheless, the effect of AC may be more evident by increasing the degradation time, as can be observed in Figure S2, and by increasing AC concentration [21,22]. Pereira et al. (2010) reported that increasing the AC concentration led to an increase of dye adsorption, favoring the electron transfer, and consequently the removal of the compounds from the medium [21]. Moreover, better reaction rates in the removal of dyes were obtained by increasing the concentration of core(ferrite, FeO)-shell(carbon, C) composites from 0.1 to 1.0 g L^−1^, once the carbon available for the electrons was more accessible for the reaction [22]. By increasing the concentration of AC, an increase in the adsorption of PFAS on its surface is also expected [29,31,32,33], thus reducing the concentration of these compounds on wastewater and adsorbed on sludge [9,18], and consequently reducing the negative impact of these compounds towards the anaerobic microorganisms. Therefore, the biodegradation of SS may occur more efficiently with the supplementation of AC.

Recent studies have reported the degradation of PFAS by single cultures of facultative *Pseudomonas parafulva*, *Pseudomonas aeruginosa* and *Pseudomonas plecoglossicida* [10,55,56]*. Pseudomonas parafulva* removed approximately 32% of PFOA after 96 h of incubation under aerobic conditions, with 67% of PFOS biologically decomposed by *Pseudomonas aeruginosa* over 48 h [10,55]. Additionally, Chetverikov et al. [56] observed degradation of PFOS by *Pseudomonas plecoglossicida*, with this strain using PFOS as a carbon source, converting it to perfluoroheptanoic acid, and releasing F^−^. Hang et al. [4] used an enrichment culture of *Acidimicrobium* sp. strain A6 for the defluorination of PFOA and PFOS. At concentrations of 100 mg L^−1^, PFOA and PFOS were the main contributors to the dissolved organic carbon, which decreased at a rate of ∼ 5.7 μM day^−1^, demonstrating that degradation of these compounds occurred.

## 3. Materials and Methods

### 3.1. Chemicals

PFOA (CAS 335-67-1, purity 95%) and PFOS (CAS 1763-23-1, ~40% in H_2_O) were obtained from Sigma-Aldrich (St. Louis, MO, USA) and sodium sulfide (Na_2_S·9H_2_O) was purchased from Fluka. All the reagents used for the preparation of the anaerobic basal medium [57] were purchased from Sigma-Aldrich and ZnSO_4_·7H_2_O was obtained from ACS (PanReac, Barcelona, Spain). All chemicals were used as received and without further purification.

### 3.2. Carbon Nanomaterials

Commercial multiwalled CNT (NC3100TM, Nanocyl SA., Sambreville, Belgium), with 1.5 μm average length, 9.5 nm average diameter, and purity > 95% of carbon, were used in the assays. AC pellets (NoritROX0.8), with 5 mm length and 0.8 mm diameter, were crushed and sieved to obtain a particle size ≤ 280 µm, to also be used as powder in the anaerobic assays. Additionally, the crushed commercial AC was submitted to an oxidative treatment in order to obtain different chemical composition on the material surface, maintaining the original textural properties of pristine AC as much as possible. Thus, an AC sample with a greater amount of oxygen-containing surface groups, and consequently stronger acid character (sample AC-HNO_3_), was obtained, as described by Pereira et al. [21]. 

Textural properties of CM, such as the specific surface area (*S*_BET_) and total pore volume (*Vp*), as well as the pH at point of zero charge (pH_PZC_), surface groups, and the results from the elemental analysis (N, C, H, S, O), are presented on Table 3 [14,15].

### 3.3. Specify Methanogenic Activity 

The effect of PFAS on AD was assessed by determining the SMA of an anaerobic granular sludge (AGS), in the presence of increasing concentrations of PFOA and PFOS, ranging from 0.1–100 mg L^−1^ (Table S2). The sludge was collected from an anaerobic digester treating wastewater from a cellulose industry. Different trophic groups were targeted by incubating the AGS with single substrates as carbon and energy sources: acetate, H_2_/CO_2_, and a mixture of volatile fatty acids (VFA), for acetoclastic and hydrogenotrophic methanogens, and acetogenic bacteria, respectively. 

Regarding acetogenic bacteria, the SMA is assessed indirectly by measuring the methane production, since the specific activity of acetogens is only directly measured when the hydrogenotrophic and the acetoclastic activities are not rate limiting [38].

SMA was determined as described by Alves et al. [58]. For the liquid substrates (acetate and VFA mixture), the biological assays were conducted in 25 mL closed serum bottles, and for the gaseous substrate H_2_/CO_2_ (80:20% *v*/*v*, at 2 × 10^5^ Pa), in bottles of 70 mL. N_2_/CO_2_ (80:20% *v*/*v*, at 2 × 10^5^ Pa) was used in the blank assay as a gaseous substrate. The working volume was 12.5 mL for all conditions (Table S2). The anaerobic medium was composed by a solution of sodium bicarbonate (3 g L^−1^) in deionized water containing resazurin (1 g L^−1^). The pH was set at 7.0 ± 0.2. The concentration of AGS was 3 g L^−1^ of volatile solids (VS). The bottles were sealed with a butyl rubber stopper and with aluminum caps, and the headspace was flushed with N_2_/CO_2_ (80:20%). Prior to the substrate addition, bottles were incubated overnight, at 37 °C under agitation at 105 rpm, in order to promote the consumption of the residual substrate. After the pre-incubation period, the batch bioreactors were flushed again with N_2_/CO_2_ (80:20%), and the substrates were added from the stock solutions to the desired concentration: acetate (30 mmol L^−1^), a mixture of VFA (10 mmol L^−1^ acetate, 10 mmol L^−1^ propionate, and 5 mmol L^−1^ butyrate), and H_2_/CO_2_ (80:20% *v*/*v*, at 2 × 10^5^ Pa). PFAS were added in concentrations ranging from 0.1 mg L^−1^ and 100 mg L^−1^ (Figure S3, Table S2). 

Initial methane (CH_4_) production rate was assessed by measuring the pressure within bottles with a pressure transducer (Paralab, Oporto, Portugal). The percentage of CH_4_ obtained from the liquid substrates consumption was analyzed by Gas Chromatography (GC), while for H_2_/CO_2_ consumption, CH_4_ production was obtained by stoichiometric calculations [58]. Blank assays without PFAS, and without substrate, were prepared, as well as control assays without PFAS but with the specific correspondent substrate. All assays were performed in triplicate.

### 3.4. Anaerobic Assays: Evaluation of the Effect of CM and PFAS on CH_4_ Production from VFA 

Following the study of the effect of PFAS on the SMA of the anaerobic communities, whether the presence of CM would alter this inhibitory effect was studied by testing different CM (CNT, AC, and AC-HNO_3_) at a concentration of 0.1 g L^−1^. These different CM were selected based on their surface characteristics, including the neutral, basic, and acid character for pristine CNT, pristine AC, and oxidized AC, respectively (Table S3). Biological assays were conducted in 70 mL serum bottles and sealed with a butyl rubber stopper, containing 25 mL of buffered medium at a pH of 7 with NaHCO_3_ (2.5 g L^−1^). The basal nutrients were: NH_4_Cl (2.8 g L^−1^), CaCl_2_ (0.06 g L^−1^), KH_2_PO_4_ (2.5 g L^−1^), and MgSO_4_.7H_2_O (1.0 g L^−1^). As primary electron donor, a mixture of VFA, containing acetate, propionate, and butyrate in a chemical oxygen demand (COD)-based ratio of 1:10:10, was added to the medium. AGS collected from an anaerobic digester treating wastewater from a cellulose industry was used as the inoculum, at a concentration of 2 g L^−1^ of VS. CM were present at a concentration of 0.1 g L^−1^, which was chosen based on previous studies [22]. The medium was flushed with N_2_/CO_2_ (80:20%) and incubated overnight at 37 °C in a rotary shaker at 105 rpm, in order to promote the consumption of the residual substrate. After the pre-incubation period, the batch bioreactors were flushed again with N_2_/CO_2_ (80:20%), and 50 mg L^−1^ of PFOA, 40 mg L^−1^ of PFOS (0.1 mmol L^−1^ PFOA; 0.1 mmol L^−1^ PFOS), and VFA (4 g L^−1^ of COD) were added (Figure S4). 

Controls were also included: blank assays without substrate in the presence and absence of CM, biological assays without CM, and abiotic assays in the presence of CM. All experiments were conducted in triplicate.

### 3.5. Toxicity Assessment with Vibrio fischeri

Evaluation of the possible toxicity of the samples after anaerobic treatment (Figure S4) was performed by the standard bioassay “Water Quality—Determination of the inhibition effect of water samples on the light emission of *Vibrio fischeri* (Luminescent bacteria test)” method, using freshly prepared bacteria [48]. *V. fischeri* strain NRRL-B-11177 was purchased as freeze-dried reagent, BioFix^®^ Lumi, from Macherey-Nagel (Düren, Germany), and grown in our laboratory under aerobic conditions [48,49,54].

The toxicity evaluation was performed based on the bacteria bioluminescence changes when exposed to potentially toxic substances. The method was adapted from the ISO 11348-1 and ISO 11348-3 standards, as described [14,15].

After the anaerobic assays to evaluate the effect of the PFAS and CM on the production of CH_4_ from VFA, the samples were centrifuged (10 min at 10,000 rpm) and filtered (Whatman SPARTAN syringe filters, regenerated cellulose, 0.2 μm pore size) prior to the toxicity assay. Negative and positive controls were prepared with the bacterial suspension: a solution of 2% NaCl and potassium dichromate (K_2_Cr_2_O_7_) at a concentration of 105.8 mg L^−1^, respectively [48,49]. The pH of all samples was measured and adjusted to values between 6 and 9 with hydrochloric acid or sodium hydroxide solutions, while the salinity was adjusted to 2% NaCl. An oxygen concentration higher than 3 mg L^−1^ was ensured and the turbidity was avoided by sample centrifugation and filtration. Luminescence inhibition (INH%) was calculated after 30 min of contact [48,49,54,59], using Equations (1) and (2):

(1) $$INH\;\left(\%\right)=100-\frac{IT_{t}}{KF\times IT_{0}}\times 100$$

With,

(2) $$KF=\frac{IC_{t}}{IC_{0}}\;$$

where *IT_t_* is the luminescence intensity of the sample after 30 min of contact; *IT*_0_ is the luminescence emission at the beginning of the assay (time 0); KF is the correction factor, which characterizes the natural loss of luminescence of the negative control; *IC_t_* is the luminescence intensity of the control after a certain the contact time (t); and *IC*_0_ is the initial luminescence intensity of the negative control. The luminescence was measured using a microplate reader (Biotek^®^ Cytation3, Fisher Scientific, Seoul, Korea) in kinetic mode and the signal recorded in relative light units (RLU Sec^−1^).

### 3.6. Anaerobic Biodegradability of Sewage Sludge Contaminated with PFAS

The anaerobic biodegradability assays of sewage sludge (SS) contaminated with PFOA and PFOS were performed according the work of Angelidaki et al. [57] and Holliger et al. [60], by evaluating the potential biochemical methane potential (BMP). The inoculum was composed by AGS, with a VS content of (0.11 ± 0.001) g g^−1^, and it was crushed to promote the homogenization of the mixture. The SS was collected from a municipal WWTP localized in the North of Portugal, and comprised (0.13 ± 0.002) g g^−1^ of VS and (0.201± 0.008) g of COD g^−1^ of sludge. The assays were performed in serum bottles of 165 mL, except for the control, where 600 mL bottles were used. The bottles were filled with the inoculum (15% *v*/*v* of the working volume) in a basal medium supplemented with micro and macro nutrients, salts, and vitamins, as described by Angelidaki et al. [57]. The medium was buffered at a pH of 7.3 ± 0.2 with NaHCO_3_ (2.5 g L^−1^). Bottles were sealed with butyl rubber stoppers and aluminum caps, and flushed with N_2_/CO_2_ (80:20% *v*/*v*). The medium was further reduced with Na_2_S·9H_2_O at a final concentration of 1 mmol L^−1^. The final working volume was 50 mL in all bottles. All assays were performed in triplicate, incubated at 37 °C, and shaken once a day (Figure S5). 

A test assay with only SS and without PFAS was performed, and also a control with additional microcrystalline cellulose (average particle size 50 μm, Acros Organic, Geel, Belgium). PFOA was added at increasing concentrations of 0.1, 1.0, 1.6, 2.0, and 3.4 g L^−1^, corresponding to 0.65, 6.5, 10.4, 12.9, and 21.8 mg of PFOA g^−1^ of SS, respectively. PFOS was added at concentrations of 0.1, 1.0, 1.2, 1.6, and 3.5 g L^−1^, corresponding to 0.52, 5.2, 7.6, 10.3, and 22.6 mg of PFOA g^−1^ of SS, respectively. The compounds’ theoretical COD were 0.270 g of COD g^−1^ of PFOA and 0.256 g of COD g^−1^ of PFOS. These values were calculated using Equation (3) [61], for compounds C*_c_*H*_h_*F*_f_*N*_n_*Na*_na_*O*_o_*P*_p_*S*_s_*:

(3) $$ThOD=\frac{16\times \left[2c+\frac{1}{2}\left(h-f-3n\right)+3s+\frac{5}{2}p+\frac{1}{2}na-o\right]\;}{MW}g/g$$

where *MW* is the molecular weight.

The effect of AC, at a concentration of 0.1 g L^−1^, on the BMP was also assessed.

The number of moles of methane produced in the headspace was obtained using Equation (4):

(4) $$n_{vial}=\frac{V_{headspacee}\;}{V_{syringe}}\times n_{sample}$$

where *n_vial_*_,_ is the number of molecules of methane in the vial headspace; *V_headspace_* is the volume (mL) of the vial headspace; *V_syringe_* is the volume of the syringe used for sampling; and *n_sample_* is the number of molecules of the sample in the syringe, at standard pressure and temperature conditions (STP) conditions [62]. 

The BMP values were obtained by dividing the volume of CH_4_ produced by the amount of VS of SS added at the beginning of the assay and are expressed in L of CH_4_ (at STP) per kg of SV of SS (L kg^−1^). The methanization percentage (MP) is obtained by dividing the obtained CH_4_ by the theoretical CH_4_, considering that 1 kg of COD of CH_4_ corresponds to 350 L of CH_4_, at STP [60].

### 3.7. Analytical Methods

The determination of the CH_4_ produced was performed by GC analysis. A gas chromatograph GC-2014 Shimadzu (Kyoto, Japan), fitted with a Porapak Q 80/100 mesh, a packed stainless-steel column (2 m × 1/8 inch, 2 mm), and a flame ionization detector (FID), was used in SMA and the anaerobic assays assessing the effect of CM and PFAS. The column, injection port, and detector temperatures were 35, 110, and 220 °C, respectively. Nitrogen was the carrier gas at a flow rate of 30 mL min^−1^.

Biomethane production from SS contaminated with PFAS was determined by GC using a GC BRUKER SCION 456 (Billerica, MA, USA) connected to a thermal conductivity detector and using a Molsieve packed column (13× 80/100, 2 m of length, 2.1 mm of internal diameter). Argon was the carrier gas at a flow rate of 30 mL min^−1^, with the temperatures of the injector, column, and detector being 100 °C, 35 °C, and 130 °C, respectively. Headspace gas was sampled using a 500 μL pressure-lock syringe (Hamilton, Reno, Nevada, EUA). The values of the cumulative CH_4_ production in the SMA and BMP assay were corrected for the standard temperature and pressure conditions (STP). A sample composed of 40:40:20% of CH_4_/CO_2_/N_2_ was used as the standard and was injected in the same GC run as the samples.

VFA consumption, in the anaerobic assays for the evaluation of the effect of CM and PFAS, was analyzed after 6 days of the experiment. The analyses were performed by HPLC (Equipment Jasco, Tokyo, Japan), using a Rezex ROA-Organic Acid H^+^ (8%) LC Column (300 × 7.8 mm), maintained at 60 °C. The mobile phase was a solution of sulfuric acid (5 mmol L^−1^), with crotonic acid used as the internal standard. The elution flow rate was 0.6 mL min^−1^ and the compounds were detected at 210 nm.

### 3.8. Statistical Analysis

Statistical significance of the differences in the anaerobic assays were evaluated by single factor analysis of variance (ANOVA) using Microsoft Excel 2016 (Office 365). Statistical significance was established at the *p* < 0.05 level.

## 4. Conclusions

PFAS are emerging recalcitrant fluorinated organic compounds, so finding an anaerobic microbial community capable of reducing PFAS is relevant to defining potential bioremediation strategies for these contaminants and obtaining a better understanding of their environmental fate.

In this work, it was found that PFAS, even at concentrations higher than those found in WWTP, did not significantly affect the activity of different trophic groups in anaerobic communities. Thus, the application of AD for the treatment of wastewater and waste contaminated with PFAS seems a promising strategy. Indeed, the sequential assays conducted suggest the feasibility of applying AD for the detoxification of wastewater and sludge containing these compounds. For instance, despite PFOA and PFOS reducing the CH_4_ production rate from the anaerobic biological conversion of VFA ((36.0 ± 5.5)% and (46.3 ± 1.6)%, respectively), this negative impact of PFOA in the CH_4_ production rate was significantly reduced by the action of CM: 15%, 2%, and 14% for PFOA, and 39%, 86%, and 36% for PFOS, with CNT, AC, and AC-HNO_3_, respectively. Furthermore, AC stimulated the AD of PFAS, increasing BMP from SS contaminated with PFOA and PFOS. Moreover, AC promoted 51% and 35% detoxification of model water samples containing PFOA and PFOS, respectively, regardless of the formed by-products, as assessed by standard *V. fischeri* assay. Based on the results, AC is the best candidate to be applied in AD to support the bioremediation of PFAS. Further, the results obtained in this work demonstrated that AD of SS contaminated with PFAS seems to be feasible and promising for application in a real context. This is because these compounds did not affect the digestion of SS in concentrations up to 0.1 and 1.2 g L^−1^, for PFOA and PFOS, respectively, which are higher than the concentrations found in a real context. On the other hand, the microorganisms were more tolerant to PFOS and it seems that this compound may potentiate the digestion of SS. In addition, AC promoted the bioconversion of SS in CH_4_ in the presence of PFOS, increasing the MP of SS in 11%.

In short, among the microbial communities present in the granular sludge used, the acetogens were the microorganisms most affected by PFAS. However, when CM are applied, the negative effect of PFAS is substantially reduced, with AC being the best-performing CM. Following, the toxicological analyses performed with the standard bioassay with *V. fischeri* to the treated samples of the anaerobic assays in the presence of CM, the results are in accordance with those obtained previously on the toxicity of PFAS towards anaerobic communities: (1) among PFAS, PFOS demonstrated the most toxic character; (2) AC was the CM that most reduced the negative impact of PFAS towards *V. fischeri*. On the other hand, when AD was applied to SS contaminated with these compounds, biodegradability was only affected by high concentrations of PFAS. Moreover, PFOS did not affect the biodegradability of the SS; instead it seemed to stimulate it, as well as AC.

The anaerobic removal of PFAS is a fairly recent topic and this work suggests, for the first time, the possibility of applying AD accelerated by the presence of small amounts of CM for the bioremediation of these recalcitrant compounds, which are problematic for the environment and public health. 

## Figures and Tables

**Table 1:** Toxicity exerted by PFOA and PFOS towards methanogenic microorganisms in the presence of different substrates.

Concentration (mg L^−1^)		Specific Methanogenic Activity Inhibition (%)		
	Acetate (30 mmol L^−1^)		VFA Mixture (10 mmol L^−1^ Acetate; 10 mmol L^−1^ Propionate; 5 mmol L^−1^ Butyrate)	H_2_/CO_2_ (80:20 % *v*/*v*, at 2 × 10^5^ Pa)
PFOA	0.1	6.6 ± 5.0	7.9 ± 4.8	0
	1	7.5 ± 4.2	11.3 ± 2.9	8.6 ± 2.6
	5	8.9 ± 3.7	16.7 ± 8.5	0
	10	12.5 ± 0.5	14.8 ± 6.5	0
	25	16.9 ± 6.0	18.7 ± 4.9	0
	50	16.0 ± 6.1	21.6 ± 2.4	0
	100	15.0 ± 1.4	25.9 ± 3.4	0
PFOS	0.1	4.4 ± 2.4	14.8 ± 5.1	0
	1	6.6 ± 5.3	20.4 ± 3.9	8.8 ± 4.4
	4	8.8 ± 3.1	22.7 ± 1.1	6.8 ± 3.5
	8	11.4 ± 6.1	24.2 ± 4.7	8.0 ± 3.1
	20	13.1 ± 1.9	24.1 ± 3.3	0
	40	23.1 ± 2.0	27.5 ± 6.4	2.5 ± 0.8
	80	24.5 ± 6.0	30.4 ± 2.4	0

**Table 2:** Methane production rate from the anaerobic degradation of VFA, in the presence of 0.1 mmol L^−1^ PFOS of PFOA and PFOS and 0.1 g L^−1^ CM.

Samples		CH_4_ Production ^a^ (L·kg^−1^·d^−1^)	
Biotic assays (AGS + VFA)	**Control without PFAS**	95.4 ± 0.6	
		**PFOA**	**PFOS**
	No CM	61.0 ± 5.2	51.2 ± 1.5
	CNT	80.8 ± 6.8	68.3 ± 4.5
	AC	94.0 ± 2.4	92.1 ± 1.2
	AC-HNO_3_	81.9 ± 2.0	68.7 ± 10.2
Blank (AGS without VFA)		0	0.9 ± 0.2

^a^ CH_4_ production (L) per kg of VS of inoculum and time (d).

**Table 3:** Results of the characterization of tested CM [14,15,21,39,47].

Sample	CNT	AC	AC-HNO_3_
S_BET_ (±10 m^2^ g^−1^)	201	1002	852
*Vp* (±0.005 cm^3^ g^−1^)	0.416	0.525	0.446
pH_PZC_ (±0.2)	6.6	8.4	4.1
Carboxylic acids (μmol g^−1^)	n.d.	110	378
Carboxylic anhydrides (μmol g^−1^)	n.d.	36	288
Carbonyl/quinones (μmol g^−1^)	n.d.	306	1130
Phenols (μmol g^−1^)	n.d.	228	815
Lactones (μmol g^−1^)	n.d.	18	88
CO (±20 μmol g^−1^)	200	598	2311
CO_2_ (±20 μmol g^−1^)	23	164	754
N (%) ^a^	0.00	0.0	1.3
C (%) ^a^	99.8	88.8	89.1
H (%) ^a^	0.11	0.4	0.9.
S (%) ^a^	0.00	0.6	0.8
O (%) ^a^	0.06	n.d.	n.d.

n.d.—Not determined; ^a^ Determined by elemental analysis.

**Table 4:** Luminescence inhibition (INH) of *Vibrio fischeri* caused by PFAS samples after 6 days of anaerobic treatment in the presence and absence of different CM.

Samples		INH (%)	
		PFOA	PFOS
Biotic assays	No CM	60 ± 0.2	53 ± 6.4
	CNT	49 ± 0.5	40 ± 6.4
	AC	31 ± 4.9	38 ± 4.4
	AC-HNO_3_	37 ± 2.5	58 ± 4.6
Abiotic assays	CNT	27 ± 7.4	30 ± 0.3
	AC	14 ± 1.9	21 ± 1.5
	AC-HNO_3_	26 ± 3.1	32 ± 2.0
Controls	PFAS	63.3 ± 0.4	58.3 ± 7.3
	Anaerobic medium	4.9 ± 0.9	
	Control (AGS + VFA)	15 ± 6.7	
	Positive control (K_2_Cr_2_O_7_)	90.8 ± 0.3	

**Table 5:** Methanization percentage (MP) and biochemical methane potential (BMP) from SS contaminated with PFOA and PFOS in the presence and absence of 0.1 g L^−1^ AC.

Samples		MP ^a^ (%, mg·mg^−1^)		BMP ^b^ (L·kg^−1^)	
		No CM	AC	No CM	AC
SS		42.0 ± 3.0	40 ± 1.0	224 ± 16	212 ± 5
SS + PFOA (g L^−1^)	0.1	41.0 ± 1.1	42.6 ± 1.4	217 ± 6	226 ± 7
	1	8.0 ± 0.3	8.9 ± 1.2	43 ± 2	47 ± 6
	1.6	5.9 ± 0.1	6.9 ± 2.7	31 ± 1	36 ± 14
	2	3.9 ± 0.3	3.6 ± 0.3	21 ± 2	20 ± 2
	3.4	3.0 ± 0.1	3.0 ± 0.4	16 ± 1	16 ± 2
SS + PFOS (g L^−1^)	0.1	42.8 ± 3.8	41.9 ± 0.9	227 ± 20	223 ± 5
	1	43.9 ± 1.7	46.0 ± 1.4	230 ± 9	244 ± 7
	1.2	52.8 ± 2.4	59.1 ± 6.2	280 ± 13	313 ± 33
	1.6	22.8 ± 1.9	22.2 ± 1.6	121 ± 10	118 ± 9
	3.5	15.8 ± 1.4	14.8 ± 4.3	84 ± 8	78 ± 23
Control (cellulose)		90 ± 3		408 ± 15	

^a^ MP—CH_4_ produced (mg) per CH_4_ theoretically expected (mg), considering that 1 kg of COD of CH_4_ corresponds to 350 L of CH; ^b^ BMP—CH_4_ produced (L) per kg of VS of SS.

## Data Availability

The data presented in this study are available in Supplementary Materials.

## Supplementary Materials

The following supporting information can be downloaded at: https://www.mdpi.com/article/10.3390/molecules27061895/s1, Figure S1: Biochemical methane potential (BMP) on the cellulose control assays (▲); Figure S2: BMP from SS + 1 g L^−1^ PFOS in the presence (●) and absence of 0.1 g L^−1^ AC (○), after 65 days of assay. SS in the presence (■) and absence of 0.1 g L^−1^ AC (□), without PFAS; Figure S3: Experimental design of the specific methanogenic activity (SMA) assays for assessing the possible toxic effect of PFAS of specific microbial trophic groups in anaerobic granular sludge (AGS) in the presence of PFOA or PFOS. VFA–volatile fatty acids; Figure S4: Experimental design of the anaerobic assays for the evaluation of the effect of PFAS and CM on CH_4_ production from VFA and toxicity assessment with *Vibrio fischeri*; Figure S5: Experimental design of the biodegradability assays of the sewage sludge (SS) contaminated with PFOA or PFOS, in the presence of activated carbon (AC); Table S1: Substrate conversion to CH_4_ over 6 days in the presence of 50 mg L^−1^ of PFOA and 40 mg L^−1^ of PFOS, and 0.1 g L^−1^ of CM. Table S2: Experimental setup of the specific methanogenic activity (SMA) assay for the different microbial trophic groups in anaerobic granular sludge (AGS) in the presence of increasing concentrations of PFAS; Table S3: Prediction of interactions between the different carbon materials (CM), anaerobic granular sludge (AGS) and PFAS, at medium pH 7.

## Sample Availability

Samples of the compounds are not available from the authors.
