# Supplementary material for: Bioremediation of Perfluoroalkyl Substances (PFAS) by Anaerobic Digestion: Effect of PFAS on Different Trophic Groups and Methane Production Accelerated by Carbon Materials
Source: Molecules. 2022 Mar 15;27(6):1895. doi: 10.3390/molecules27061895 (PMC8952860; doi:10.3390/molecules27061895)
Supplement: Supplementary file 1 [file molecules-27-01895-s001.zip › molecules-1581735-supplementary.pdf]

Supplementary information

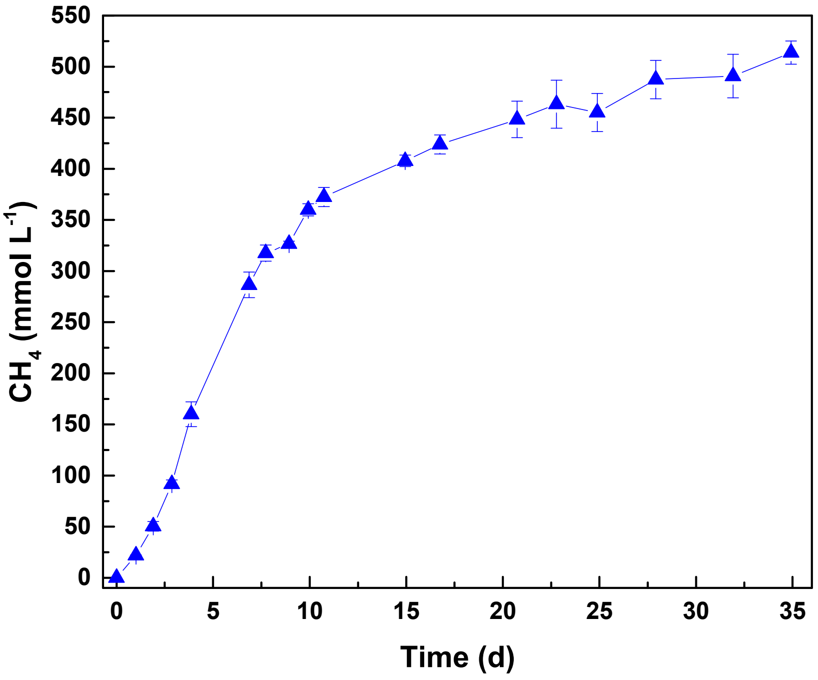

Figure S1. Biochemical methane potential (BMP) on the cellulose control assays (▲).

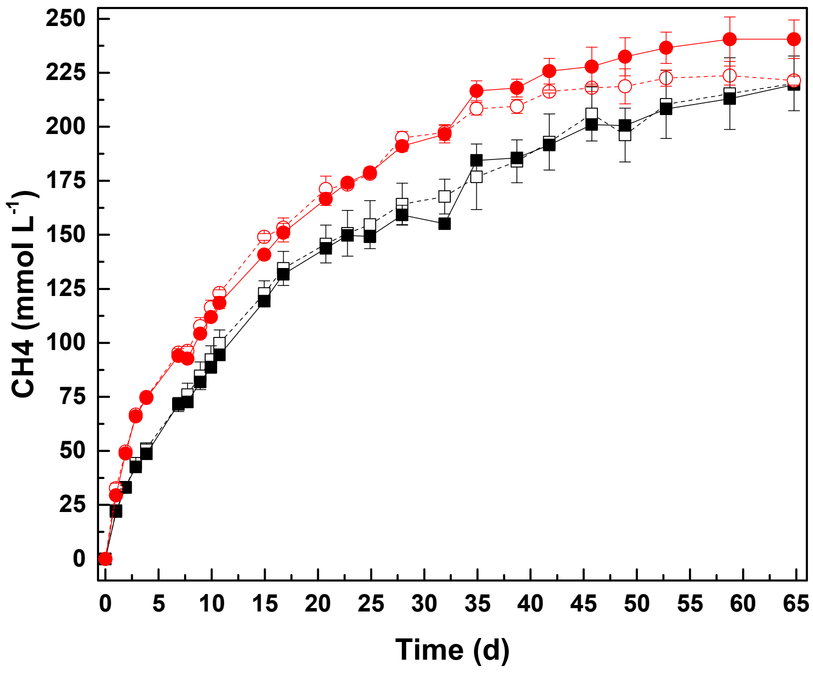

Figure S2. BMP from SS+1 g L^{-1} PFOS in the presence (●) and absence of 0.1 g L^{-1}AC (○), after 65 days of assay. SS in the presence (■) and absence of 0.1 g L^{-1}AC (□), without PFAS.

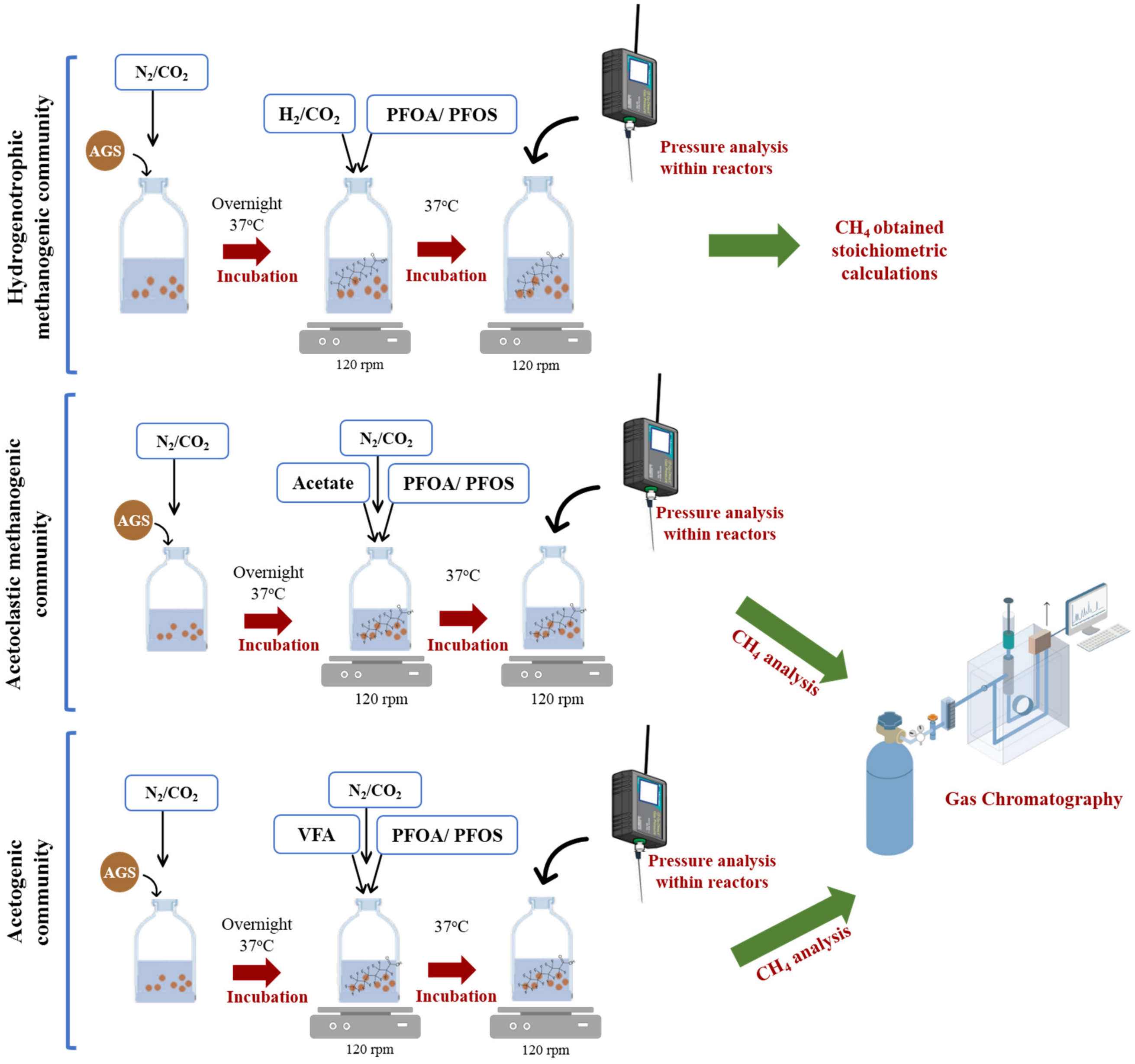

Figure S3. Experimental design of the specific methanogenic activity (SMA) assays for assessing the possible toxic effect of PFAS of specific microbial trophic groups in anaerobic granular sludge (AGS) in the presence of PFOA or PFOS. VFA – volatile fatty acids.

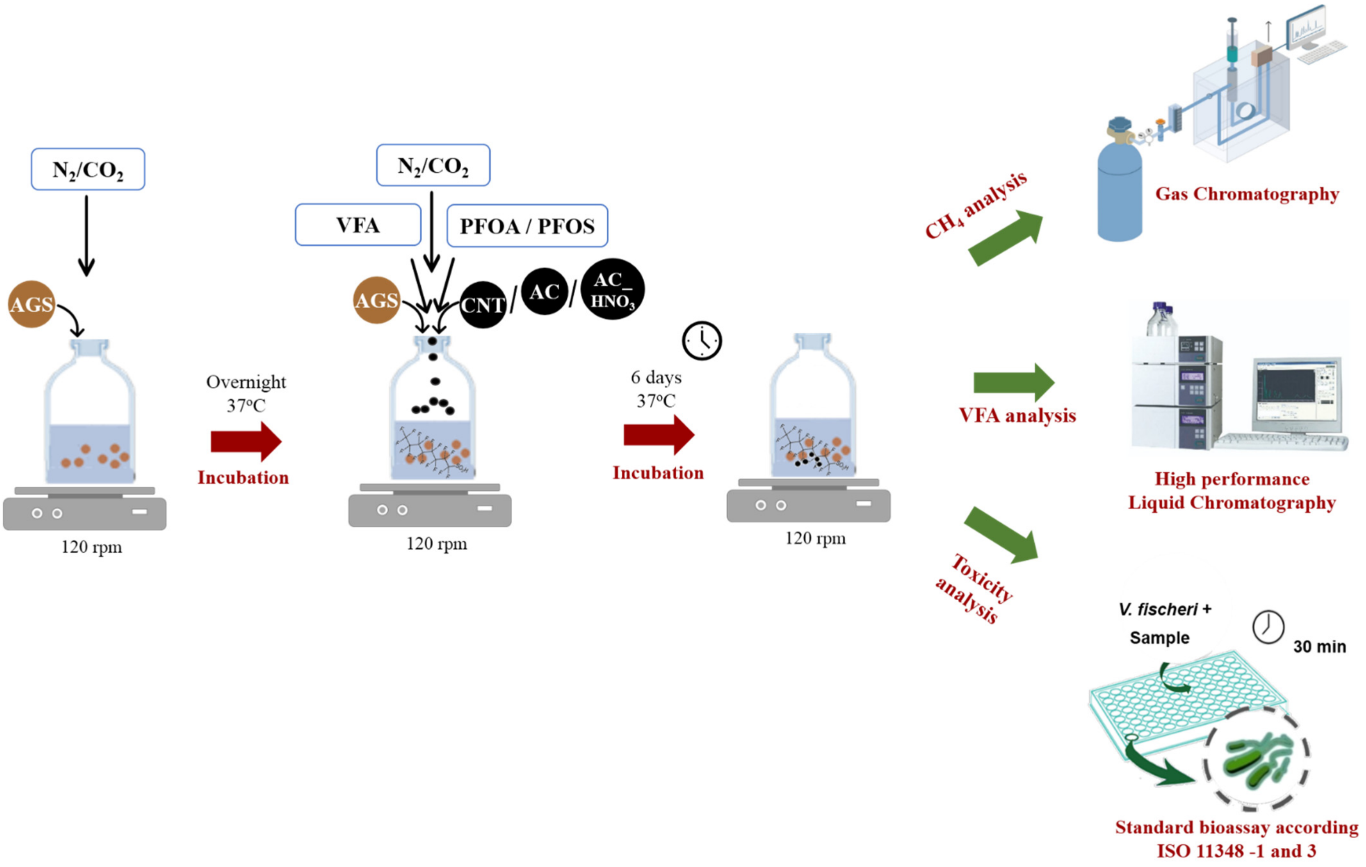

Figure S4. Experimental design of the anaerobic assays for the evaluation of the effect of PFAS and CM on CH_4 production from VFA and toxicity assessment with *Vibrio fischeri.*

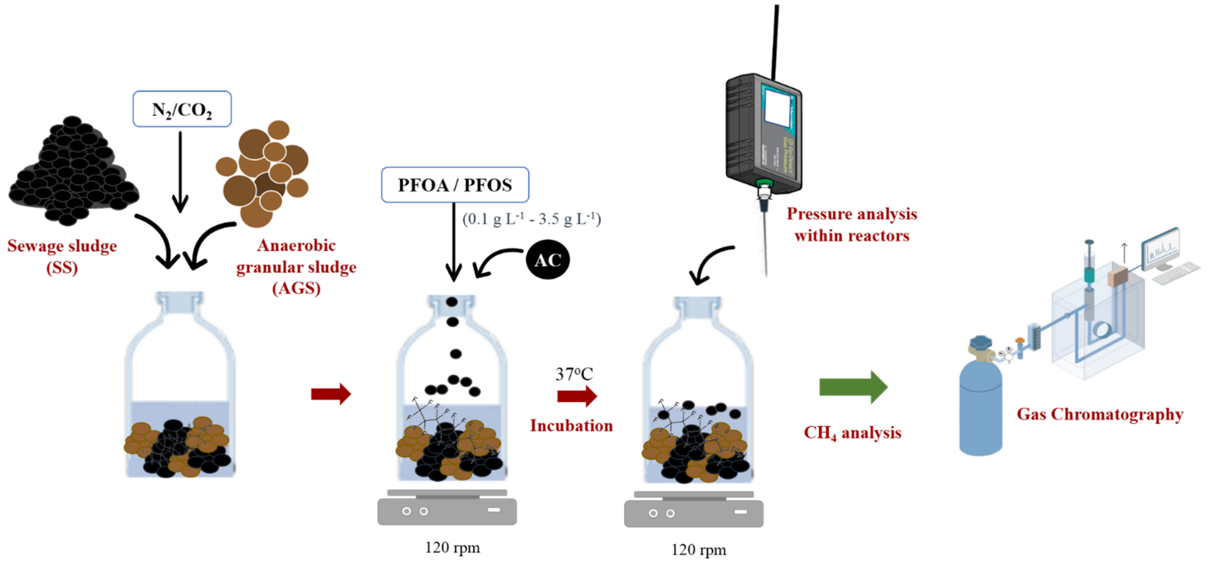

Figure S5. Experimental design of the biodegradability assays of the sewage sludge (SS) contaminated with PFOA or PFOS, in the presence of activated carbon (AC).

Table S1. Substrate conversion to CH_4 over 6 days in the presence of 50 mg L^{-1} of PFOA and 40 mg L^{-1} of PFOS, and 0.1 g L^{-1} of CM

	Sample	Acetate (mmol L^{-1})		Propionate (mmol L^{-1})		Butyrate (mmol L^{-1})		CH_4 (mmol L^{-1})	
		**t=0**	**t=6 days**	**t=0**	**t=6 days**	**t=0**	**t=6 days**	**Expected from VFA conversion**	**Obtained**
	Control (AGS+VFA)	2.9 ± 0.2	0	18.3 ± 0.9	0	12.1 ± 0.4	0	65.2 ± 2.8	61.2 ± 1.3
**PFOA**	AGS+VFA+PFOA	2.8 ± 0.2	0.7 ± 0.8	17.7 ± 1.4	0	12.0 ± 0.8	0.8 ± 1.1	61.1 ± 1.3	55.8 ± 5.0
	AGS+VFA+PFOA +CNT	2.9 ± 0.1	0	19.2 ± 1.5	0	12.2 ± 0.1	0	67.0 ± 2.3	59.8 ± 1.2
	AGS+VFA+PFOA +AC	2.8 ± 0.1	0	14.3 ± 1.8	0	12.2 ± 0.8	0	58.3 ± 5.2	60.7 ± 1.2
	AGS+VFA+PFOA +AC_HNO_3	2.7 ± 0.1	0	16.9 ± 1.0	0	11.8 ± 0.8	0	61.7 ± 3.9	60.6 ± 3.5
**PFOS**	AGS+VFA+PFOS	2.7 ± 0.1	1.6 ± 0.1	17.1 ± 0.2	0	12.0 ± 0.1	6.7 ± 1.4	43.8 ± 2.9	39.1 ± 0.9
	AGS+VFA+PFOS +CNT	2.6 ± 0.1	0	16.0 ± 1.0	0	11.2 ± 0.1	0	56.3 ± 1.6	59.4 ± 2.2
	AGS+VFA+PFOS +AC	2.9 ± 0.2	0.1 ± 0.1	17.0 ± 1.0	0	8.4 ± 2.0	0	58.3 ± 1.6	61.1 ± 0.8
	AGS+VFA+PFOA + AC_HNO_3	2.7 ± 0.2	1.07 ± 0.1	16.2 ± 1.3	0	10.7 ±0.8	0.2 ± 0.1	56.2 ± 4.4	58.2 ± 5.1

Table S2. Experimental setup of the specific methanogenic activity (SMA) assay for the different microbial trophic groups in anaerobic granular sludge (AGS) in the presence of increasing concentrations of PFAS

Condition	Concentration (mg L^{-1})	AGS (g L^{-1} VS)	Substrate		
			Acetate (mmol L^{-1})	VFA mixture (mmol L^{-1})	H_2/CO_2
**Control**	n.a.	3	30	Acetate :10<br>Propionate:10<br>Butyrate: 5	80:20 % v/v, at $2x10^5$ Pa
**PFOA**	0.1; 1; 5; 10; 25; 50; 100				
**PFOS**	0.1; 1; 4; 8; 20; 40; 80				
**Blank**	n.a.		n.a.	n.a.	N_2/CO_2 (80:20 % v/v), at $2x10^5$ Pa
		**Working volume (mL)**	12.5	12.5	12.5
		**Headspace volume (mL)**	12.5	12.5	57.5

Table S3. Prediction of interactions between different materials of carbon (CM), anaerobic granular sludge (AGS), and PFAS, at medium pH 7

CM			Interactions			References
	pH_{PZC}	Charge	AGS (-)	PFOA (-)	PFOS (-)	
**CNT**	6.6	Neutral	Neutral	Neutral	Neutral	[1–5]
**AC**	8.4	(+)	Attraction	Attraction	Attraction	[1–3,5,6]
**AC_HNO$_3$**	4.1	(-)	Repulsion	Repulsion	Repulsion	[1–3,5,6]
